# Heterogeneity and Remodeling of Ion Currents in Cultured Right Atrial Fibroblasts From Patients With Sinus Rhythm or Atrial Fibrillation

**DOI:** 10.3389/fphys.2021.673891

**Published:** 2021-06-03

**Authors:** Dorothee Jakob, Alexander Klesen, Elisa Darkow, Fabian A. Kari, Friedhelm Beyersdorf, Peter Kohl, Ursula Ravens, Rémi Peyronnet

**Affiliations:** ^1^Institute for Experimental Cardiovascular Medicine, University Heart Center Freiburg – Bad Krozingen, Freiburg, Germany; ^2^Medical Center and Faculty of Medicine, University of Freiburg, Freiburg, Germany; ^3^Spemann Graduate School of Biology and Medicine (SGBM), University of Freiburg, Freiburg, Germany; ^4^Faculty of Biology, University of Freiburg, Freiburg, Germany; ^5^Department of Cardiovascular Surgery, University Heart Center Freiburg – Bad Krozingen, Freiburg, Germany; ^6^CIBSS Centre for Integrative Biological Signaling Studies, University of Freiburg, Freiburg, Germany

**Keywords:** voltage-gated channels, BK_*Ca*_, heart, fibrosis, proliferation

## Abstract

Cardiac fibroblasts express multiple voltage-dependent ion channels. Even though fibroblasts do not generate action potentials, they may influence cardiac electrophysiology by electrical coupling *via* gap junctions with cardiomyocytes, and through fibrosis. Here, we investigate the electrophysiological phenotype of cultured fibroblasts from right atrial appendage tissue of patients with sinus rhythm (SR) or atrial fibrillation (AF). Using the patch-clamp technique in whole-cell mode, we observed steady-state outward currents exhibiting either no rectification or inward and/or outward rectification. The distributions of current patterns between fibroblasts from SR and AF patients were not significantly different. In response to depolarizing voltage pulses, we measured transient outward currents with fast and slow activation kinetics, an outward background current, and an inward current with a potential-dependence resembling that of L-type Ca^2+^ channels. In cell-attached patch-clamp mode, large amplitude, paxilline-sensitive single channel openings were found in ≈65% of SR and ∼38% of AF fibroblasts, suggesting the presence of “big conductance Ca^2+^-activated K^+^ (BK_*Ca*_)” channels. The open probability of BK_*Ca*_ was significantly lower in AF than in SR fibroblasts. When cultured in the presence of paxilline, the shape of fibroblasts became wider and less spindle-like. Our data confirm previous findings on cardiac fibroblast electrophysiology and extend them by illustrating differential channel expression in human atrial fibroblasts from SR and AF tissue.

## Introduction

Atrial fibrillation (AF) is a common arrhythmia of increasing prevalence due to an aging population (Chugh et al., 2014). AF is associated with increased morbidity and mortality, and long-term success of current treatment options is limited. One of the many reasons for the unsatisfactory outcome of pharmacological or interventional therapies is thought to be related to the progressive nature of the arrhythmia (Jahangir et al., 2007). As AF proceeds from initial, self-terminating episodes to permanent AF, the atria undergo structural and electrical remodeling, including fibrosis and electrophysiological changes (Quah et al., 2021). Whilst fibroblasts are clearly involved in the former process, much less is known about their role in the latter.

Fibroblasts are essential for cardiac tissue repair and maintenance of mechanical stability of the heart (Souders et al., 2009). They are able to sense and adapt to a variety of mechanical and chemical signals involved in stress and injury responses. Activated upon tissue damage, fibroblasts proliferate, and *trans-*differentiate into myofibroblasts that migrate toward the lesion and secrete high amounts of fibrillar collagen, for example during scar formation (Camelliti et al., 2005). Excessive collagen accumulation may also lead to fibrosis in non-lesioned tissue, a process which contributes to AF by increasing mechanical and electrical heterogeneity (Krul et al., 2015). In addition, cardiac fibroblasts (Quinn et al., 2016; Rubart et al., 2018), as well as other non-myocytes such as immune cells (Hulsmans et al., 2017), may modify the electrophysiology of cardiomyocytes through direct electrically conductive contacts. Electrotonic interactions of the two cell types will depolarize cardiomyocytes (due to the less negative resting membrane potential of non-myocytes), and potentially slow conduction [due to the addition of a passive electrical load (Kohl et al., 2005)], which could promote re-entry.

Cardiac fibroblasts do not generate action potentials, though they express multiple voltage-, ligand- and mechano-dependent ion channels (Shibukawa et al., 2005; Kamkin et al., 2010; Benamer et al., 2013). Among the ion currents described in cultured human cardiac fibroblasts are Na^+^ currents, various K^+^ currents, current conducted *via* BK_*Ca*_ channels, and Cl^–^ currents (Wang et al., 2006; Li et al., 2009; Kamkin et al., 2010; Chatelier et al., 2012; Poulet et al., 2016; Klesen et al., 2018); a more comprehensive listing of currents and references is given in the Supplementary Table 1. Interestingly, in a canine model of AF, K^+^ currents of atrial fibroblasts were altered, compared to cells from non-fibrillating atria (Wu et al., 2014; Qi et al., 2015). Also, human atrial fibroblasts undergo phenotypic changes with AF (Poulet et al., 2016). The objective of the present study was to assess and extend previous observations, in order to provide insight into changes in non-myocyte electrophysiology that may contribute to the complex pathophysiology of AF.

## Materials and Methods

### Tissue Samples and Patient Demographics

All patients gave written and informed consent prior to inclusion into the study, and investigations conformed to the principles outlined in the Declaration of Helsinki. Tissue pieces were excised from the right atrial appendage as a routine procedure in the course of cannulation for extracorporeal circulation during open heart surgeries. Excised tissue samples were placed in room-temperature cardioplegic solution [containing in (mmol L^–1^): NaCl 120, KCl 25, HEPES 10, glucose 10, MgCl_2_ 1; pH 7.4, 300 mOsm L^–1^] and immediately transported to the laboratory, where they were processed by the Cardiovascular Biobank at the University Heart Center Freiburg Bad Krozingen (approved by the ethics committee of Freiburg University, No 393/16; 214/18). Patients were either in sinus rhythm (SR), or had sustained AF [which includes patients with persistent, long-standing persistent and permanent AF, defined according to ESC Guidelines (Hindricks et al., 2021)]. Patient demographics are listed in Table 1.

**TABLE 1:** Patient characteristics.

		**SR**	**AF^1)^**	***p***
Number of patients (male/female)		7 (4M/3F)	4 (2M/2F)	
Age at time of surgery (years)		66.6 ± 9.1^2)^	71.1 ± 8.4	0.297
ASA Stage		3.4 ± 0.5	3.2 ± 0.5	0.651
BMI (kg m^–2^)		26.0 ± 4.6	23.5 ± 2.9	0.636
Diabetes mellitus		1	0	
Hyperlipidemia		1	1	
Arterial hypertension		3	1	
Blood pressure (mmHg)	Systolic	126.2 ± 10.2	115.0 ± 17.3	0.439
	Diastolic	73.3 ± 13.3	51.3 ± 10.3	**0.036**
Heart rate		83.1 ± 23.6	83.3 ± 13.6	0.849
Ejection fraction (%)		43.0 ± 13.5	48.5 ± 4.7	0.458
Surgical procedures				
Aorto-coronary venous bypass		3	1	
Aortic valve replacement/reconstr.		3	3	
Mitral valve replacement/reconstr.		2	4	
Pulmonary valve repl./reconstr.		0	0	
Tricuspid valve repl./reconstr.		0	0	
Heart transplantation		1	0	
Medication				
ACE Inhibitors		4	1	
ATI-receptor blocker		0	1	
β-blocker		4	3	
Diuretics		2	2	
Aldosteron antagonists		0	0	
Nitrates		0	0	
Statins		2	3	
Anticoagulants		4	3	

*^1)^AF – sustained AF defined according to ESC Guidelines, including patients with persistent, long-standing persistent and permanent AF (Hindricks et al., 2021).^2)^Mean ± SD.*

### Fibroblast Isolation and Culture

In order to obtain fibroblasts by the “outgrowth technique,” tissue was processed within 30 min of excision, as described previously (Poulet et al., 2016). In brief, the samples (50–200 mg) were cut into small chunks of roughly 1 mm edge length and transferred into a 6-well plate. Each well contained 2 mL of Dulbecco’s modified Eagle medium (Thermo Fisher Scientific, Germany), 10% fetal calf serum, and 1% penicillin-streptomycin (all Sigma-Aldrich, Germany), for incubation at 37°C in an atmosphere of air supplemented with CO_2_ to maintain 5% CO_2_. Culture medium was changed twice a week. Cells migrated from the tissue chunks, proliferated and reached ≈80% confluency after 20–28 days. Then, after washing with Dulbecco’s phosphate-buffered saline (Sigma Aldrich, Germany), cells were detached with 0.05% trypsin (trypsin-EDTA solution; Sigma Aldrich). The suspension was centrifuged (57 × *g*, 3 min; Rotina 380, Hettich, Germany), the supernatant removed, and the pellet re-suspended in the cell culture medium described above. Cells were re-seeded for subculture in tissue culture flasks or for experiments in uncoated Ø 35 mm plastic dishes (93040, Techno Plastic Products, Germany). Only cells from passage 0 and 1 were used for experimentation (*i.e.*, from 3 to 6 weeks post isolation). The cells had typical spindle-like or polygonal shapes (Figure 5C), as described in earlier work (Poulet et al., 2016; Klesen et al., 2018). They formed a mixed population of vimentin-positive fibroblasts and αSMA-positive myofibroblasts (82% and 18% respectively; data not shown).

**FIGURE 1:**
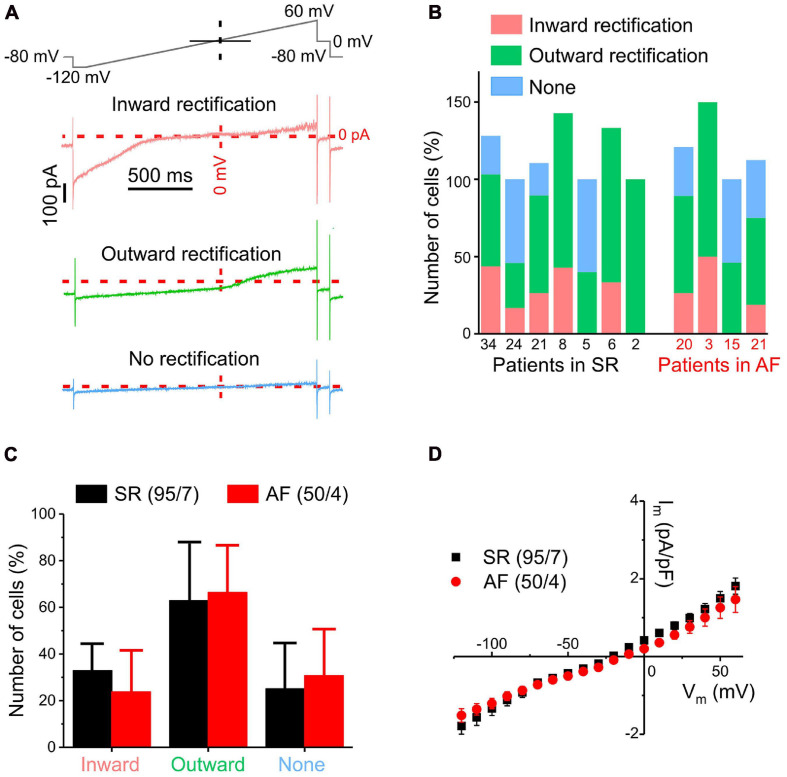
Overview of different ion current patterns in human atrial fibroblasts recorded using a ramp pulse protocol in whole-cell mode. **(A)** Exemplary traces of different current types (inward rectification, outward rectification, no rectification) with the ramp protocol used (top trace). **(B)** Distribution of current types between cells from different patients with SR or AF. Note that percentages may add up to >100% because some cells show both inward and outward rectification; the number of cells assessed for each patient is indicated below the x-axis. **(C)** Distribution of current types between cells from SR and AF patients (error bar = standard deviation between patients). **(D)** Pooled current/voltage (IV) curves of all cells from patients with SR (black) or AF (red): Both IV curves have a reversal potential of approximately –20 mV. No statistically significant differences in current density between cells from SR and AF patients were observed.

**FIGURE 2:**
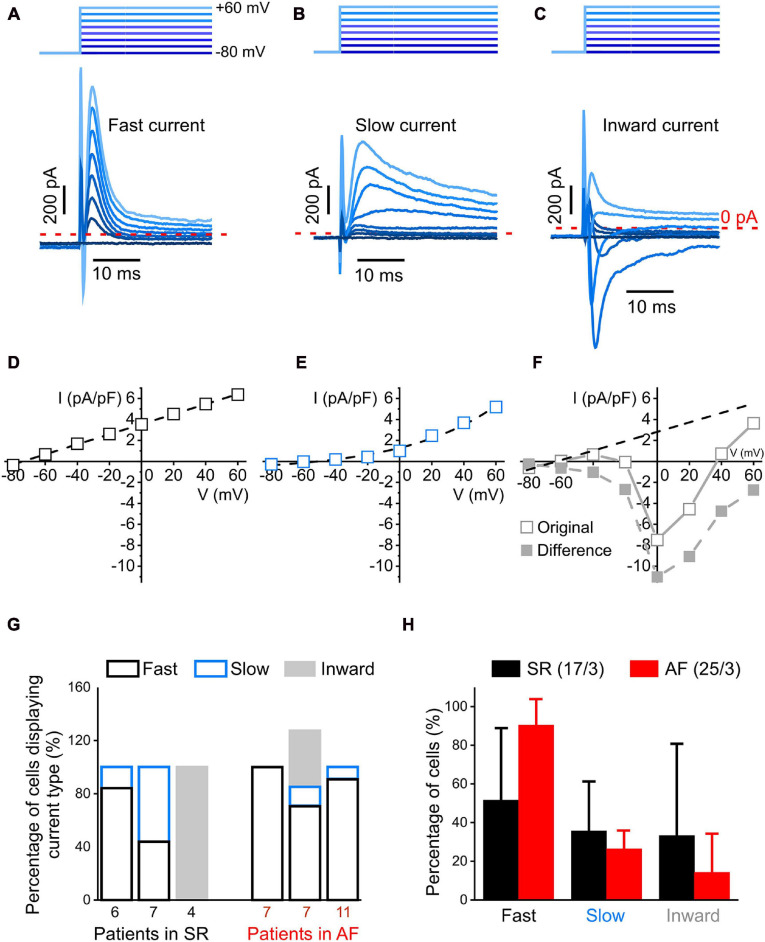
Currents profiles with fast kinetics identifiable with a step-pulse protocol (whole-cell mode). **(A–C)**: Representative recording of three different current profiles measured during the first 30 ms after depolarization: rapidly activating and inactivating transient outward current (fast current, **(A)**, more slowly activating and inactivating outward current [slow current, **(B)**] and inward current observed at holding potentials ranging from –20 to +60 mV **(C)**. 1 s-long voltage clamp pulses were used to depolarize cells from –80 to +60 mV in steps of 20 mV; the holding potential used between pulses was –80 mV (top schematic). **(D–F)** Representative IV curves of peak current. In panel **(F)**, the difference current IV curve was obtained by subtracting from the original IV curve the one of the fast current **(D)**. **(G)** Distribution of current profiles within cells from three patients each with SR and AF. Note that percentages may add up to >100% because some cells presented two current activation patterns. **(H)** Comparison of the percentage of cells exhibiting fast and slow outward, and inward current in all cells analyzed from patients with SR and AF. No statistically significant differences in the distribution of current profiles between cells from SR and AF patients were observed.

**FIGURE 3:**
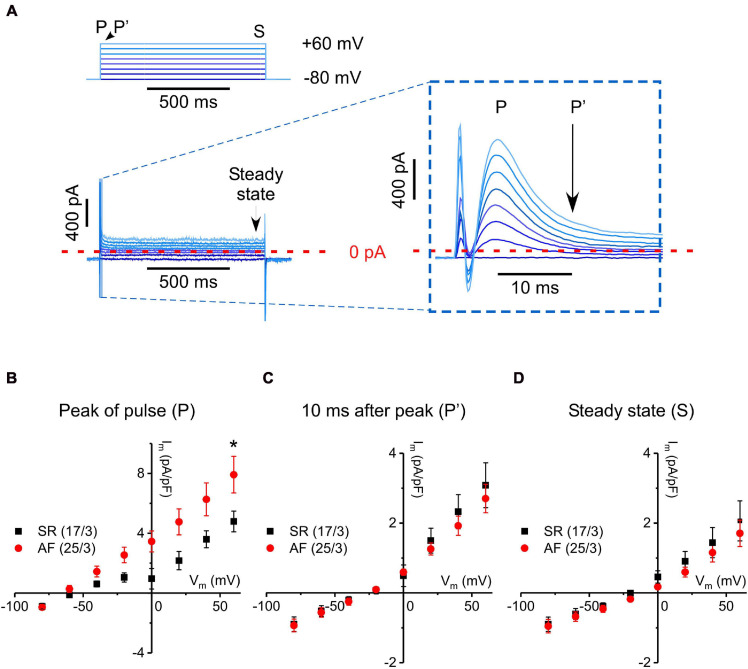
Fast transient outward current measured with a step-pulse protocol in cells from tissue donors with SR and AF. **(A)** Pulse protocol and a representative current recording. Inset: Enlargement of early (first 10–15 ms) current dynamics, as indicated in A (stimulation artifact truncated in the enlarged image). Depolarizing steps activate a linearly increasing, transient current of <10 ms in duration. **(B–D)** IV curves constructed from peak current values (P), from values 10 ms after P (P′), and from values at the end of each voltage step (S, steady state), respectively, for fibroblasts from SR and AF donors. Please note different scales of Y-axes. *Indicates significant difference.

**FIGURE 4:**
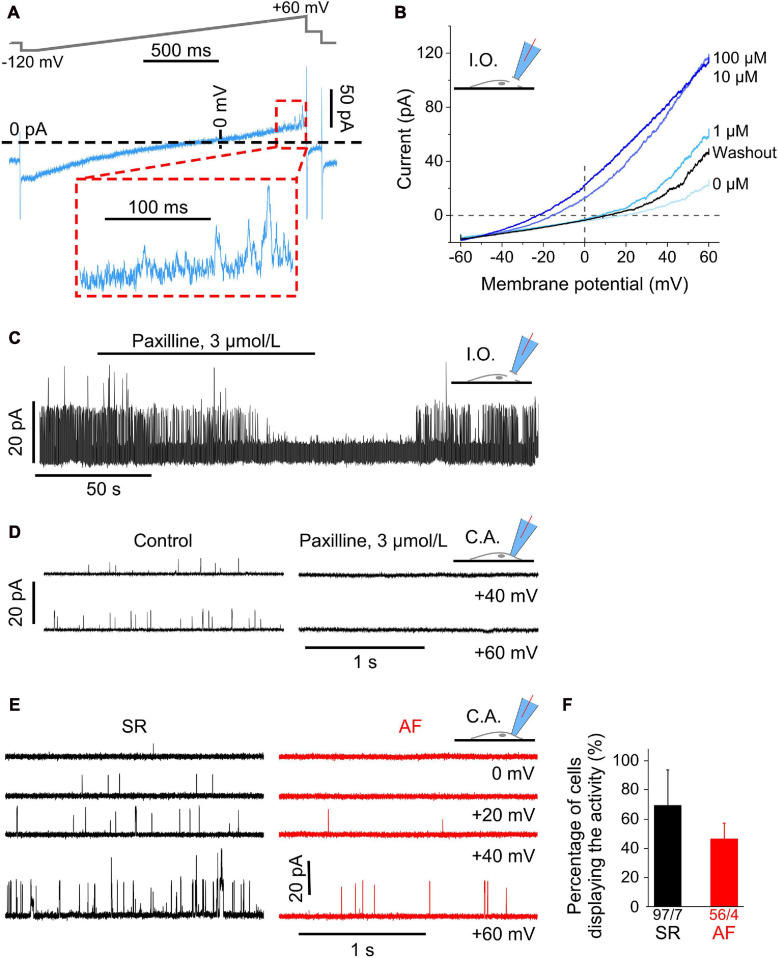
Large Ca^2+^-dependent conductance activated in the positive potential (outward current) range in human atrial fibroblasts. **(A)** Representative current trace measured with the ramp pulse protocol (whole-cell recording, ruptured patch mode). The inset shows the final 200 ms of the trace (outward current) at positive potentials; the current displays clear single channel openings. **(B)** Voltage-dependent current amplitude at different intracellular Ca^2+^ concentrations (from 0 to 0.1 mM), and after returning to nominally Ca^2+^-free solution (washout). Recordings obtained in inside-out configuration (I.O.). **(C)** Channel activity in an inside-out patch transiently superfused with paxilline-containing solution. Note that the paxilline-induced inhibition of channel activity was partially reversed during wash-out. **(D)** Single channel activity in cell-attached patches without (control) and with 3 μmol L^− 1^ paxilline in the pipette solution. **(E)** Single channel events during representative current traces at various holding potentials (cell-attached patch mode) in a cell from an SR and AF patient. **(F)** Percentage of cells in relation to all cells studied per patient (see total numbers below the columns) that exhibit single channel openings during the final 200 ms of the ramp protocol.

**FIGURE 5:**
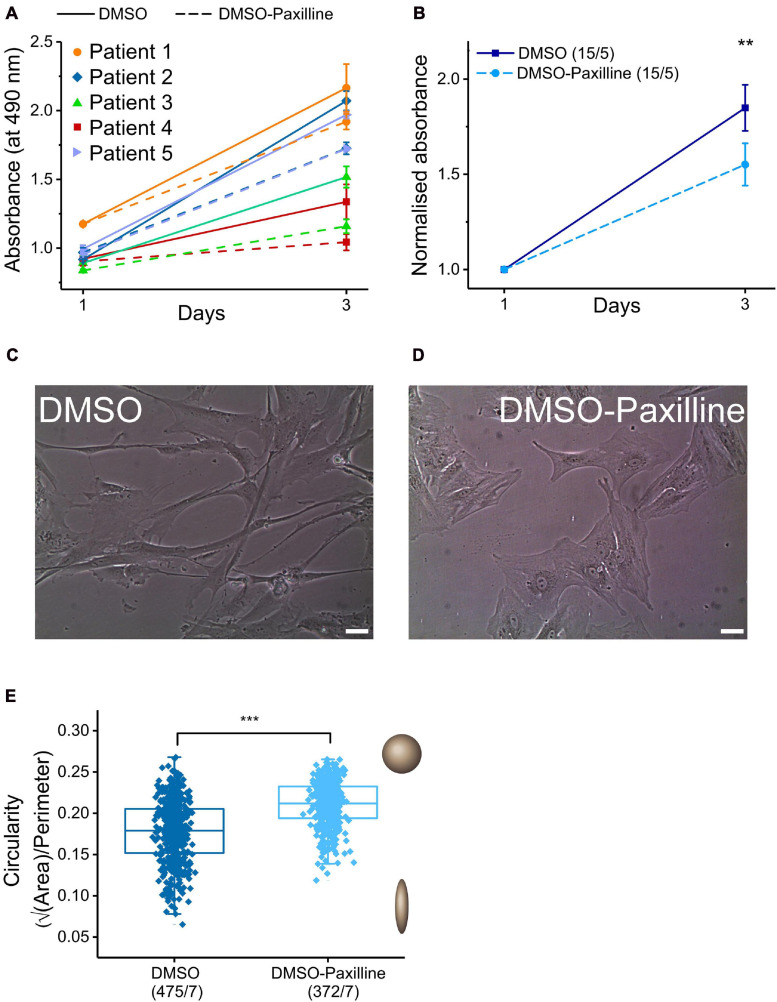
Effects of BK_*Ca*_ channel blocker paxilline on proliferation rate and cell shape. **(A)** Proliferation assay in the presence (dashed lines) and absence (continuous lines) of the BK_*Ca*_ channel blocker paxilline (10 μmol L^− 1^) after 1 and 3 days in matched samples from five individual SR patients (triplicate measurements for each point). **(B)**: Mean values for results from the proliferation assay. When normalized to the absorbance value of day 1, the mean increase in absorbance of cells incubated with paxilline was lower (1.6 ± 0.077) than for control conditions (1.8 ± 0.077). **(C,D)** Representative images of fibroblasts cultured for 2 days with and without paxilline. Scale bar = 20 μm. **(E)** Comparison of cell-shape (circularity) of cells incubated with DMSO (control) *vs*. 10 μmol L^− 1^ paxilline in DMSO.

### Patch-Clamp Technique

Ion currents or single channel activity in primary cultures of atrial fibroblasts were measured by the patch-clamp technique, either in ruptured patch whole-cell or in cell-attached mode, respectively. Micropipettes were pulled from fire-polished soda-lime glass capillaries (inner diameter: 1.15 ± 0.05 mm, outer diameter: 1.55 ± 0.05 mm; VITREX Medical, Denmark) with a standard pipette puller (PC-10, Narishige, Japan), pipette resistance was 1–3 MΩ when filled with pipette solution for whole-cell recordings, containing (in mmol L^–1^): NaCl 8, KCl 40, K-aspartate 80, CaCl_2_ 2, Tris-GTP 0.1, Mg-ATP 5, EGTA 5, HEPES 10 (adjusted to pH 7.4 with KOH), ≈300 mOsm L^–1^. The bath solution contained (in mmol L^–1^): NaCl 150, KCl 5.4, CaCl_2_ 2, HEPES 10, glucose 5 (adjusted to pH 7.4 with NaOH), ≈300 mOsm L^–1^.

For cell-attached recordings, the pipette solution contained (in mmol L^–1^): NaCl 150, KCl 5, CaCl_2_ 2, HEPES 10 (adjusted to pH 7.4 with NaOH), ≈300 mOsm L^–1^ and the bath solution (in mmol L^–1^): KCl 155, EGTA 5, MgCl_2_ 3, and HEPES 10 (adjusted to pH 7.2 with KOH). Patch excision, to obtain the inside-out mode, was achieved by a fast upward displacement of the patch pipette. Experiments were performed at room temperature (≈20°C), using a patch-clamp amplifier (200B, Axon Instruments, United States) and a Digidata 1440A interface (Axon Instruments). Recorded signals were digitized at 3 kHz, low-pass filtered at 1 kHz, and analyzed with pCLAMP10.3 software (Axon Instruments) and Origin9.1 (OriginLab, United States). Membrane capacitance was measured using fast depolarizing ramp pulses (−55 to −50 mV, 5 ms) after gigaseal formation and breaking the cell membrane. At passage 0 and 1, no statistically significant differences in membrane capacitance were detected between cells from SR and AF patients (Supplementary Figure 1).

#### *KCNMA1* mRNA Expression

To determine mRNA expression levels relative to glyceraldehyde 3-phosphate dehydrogenase (*GAPDH*) by quantitative polymerase chain reaction (qPCR), mRNA isolation from immortalized human atrial fibroblasts (Künzel et al., 2020) was performed using a commercial RNA isolation kit (RNeasy Micro Kit; Qiagen, Germany). Complementary DNA was amplified in TaqMan Fast Advanced Master Mix (4444556, ThermoFisher) for a total of 40 cycles. A more detailed protocol can be found in Emig et al. (2021).

*KCNMA1* expression in primary atrial fibroblasts from patients with SR and AF was assessed based on the Affymetrix GeneChip array (Affymetrix, Santa Clara, CA, United States) analysis described in Poulet et al. (2016). Briefly cells were cultured for 3 weeks, replated at a density of 2.5 × 10^3^ cells per cm^2^ and kept two more weeks in culture before analysis. Hybridization at 45°C and 60 revolutions per minute for 16 h (hybridization oven 640; Affymetrix), staining and washing, processing (Fluidics Station 450, Affymetrix) and scanning (GeneChip Scanner 3000 G7) were all carried out as recommended by Affymetrix.

### Proliferation Assay

Cells were seeded at a density of 5,000 cells per well in a 24-well plate with the culture medium described above. Cell proliferation was assessed using the colorimetric CellTiter 96^®^ Aqueous One Solution Cell Proliferation Assay (Promega, United States). The solution contained the tetrazolium compound MTS [3-(4,5-dimethylthiazol-2-yl)-5-(3-carboxymethoxyphenyl)-2-(4-sulfophenyl)-2H-tetrazolium]. When MTS was added to cultures for 60 min in the incubator, viable cells converted the compound into a colored formazan dye, measured spectrometrically at 490 nm using a Biospectrometer^®^ Basic (Eppendorf, Germany). The concentration of formazan is proportional to metabolic activity and to the number of viable cells.

### Cell Shape

In order to quantify changes in cell shape, we analyzed individual cell images acquired by an inverted microscope Nikon Eclipse TS100 (Nikon, Japan) for cell area (*A*) and cell perimeter (*P*). For a perfect circular shape, the radius calculated from cell area (*r*_*A*_) is equal to the radius calculated from cell perimeter (*r*_*P*_), *r*_*A*_ = *r*_*P*_. Given *r*_*A*_ = √*A*/√π (with *A* = π*r*^2^) and *r*_*P*_ = *P*/2π (with *P* = 2π*r*), √*A*/*P* = √π/2π, and √*A*/*P* = 0.2821. Image procession and analysis were conducted with ImageJ (Schindelin et al., 2012; Schneider et al., 2012).

### Statistical Analysis

Graphs and statistical analyses were generated using Origin9.1 (OriginLab Corporation, United States). For normally distributed data (Shapiro-Wilk test), the unpaired two-tailed Student’s *t*-test was used. For *n* < 25 or not normally distributed data, the Mann–Whitney test was used. Results were considered as indicative of a significant difference between means if *p* < 0.05. Asterisks in figures indicate the following *p*-values: ^∗^*p* < 0.05, ^∗∗^*p* < 0.01, ^∗∗∗^*p* < 0.001.

## Results

### Steady-State Currents, Whole-Cell Mode

Voltage ramp protocols lend themselves to the study of steady-state currents because if membrane potential changes are slow enough, rapid transient currents will already be inactivated and therefore not visible. Voltage-dependent currents, measured during a 2 s-long ramp pulse from −120 to +60 mV (rate of voltage change 90 mV s^–1^, top trace), exhibited three different rectification patterns in fibroblasts from both patient groups: inward, outward or no rectification (Figure 1A). Figure 1B illustrates the distribution of these types of rectification between cells from each of the seven SR and four AF patients studied. The numerals below each column indicate the number of cells assessed for each patient; the distribution is expressed in percent of fibroblasts exhibiting a particular type of rectification, noting that some cells showed both inward and outward rectification. The inter-patient variability of percentage of cells exhibiting any particular type of rectification was large and no statistically significant differences in distribution of current types between fibroblasts from patients with SR and AF were detected (Figure 1C). In addition, the mean current/voltage relationships (IV-curves), obtained from all cells studied, were not statistically different in fibroblasts from either group (Figure 1D).

### Voltage- and Time-Dependent Currents, Whole-Cell Mode

In order to study the voltage dependence of ion channels with fast kinetics, 1 s-long voltage clamp pulses were used to depolarize cells from −80 to +60 mV in steps of 20 mV. During the initial 30 ms of the clamp steps, we observed three different profiles of transient currents: a time-dependent outward current with fast activation and inactivation (“fast current,” Figure 2A), a time-dependent outward current with slow inactivation kinetics (“slow current,” Figure 2B), and a current that had a net inward component at depolarization steps to 0 and +20 mV (“inward current,” Figure 2C). The IV-curve for the fast transient outward current was linear with a reversal potential near −70 mV, while the IV-curve for the slow transient outward current was exponential with a reversal potential near −50 mV (Figures 2D,E). The inward current (Figures 2C,F “original”) appeared superimposed with a fast outward current with a linear voltage-dependence. The extrapolated linear IV-curve between −80 and +40 mV had a conductance of 31 pS, compared to the fast current conductance of 48 pS (Figure 2D). Subtracting this fast current from the recorded inward current yielded the IV-curve shown in Figure 2F, “difference.” The inward current component was observed in 12% of AF cells (*n* = 3 of 25) and 24% of SR cells (*n* = 4 of 17). Comparing three patients each for AF and SR, there were no statistically significant differences in the number of cells showing any of these three current patterns (Figures 2G,H).

The fast transient outward current, i.e., the most frequently seen current type, was analyzed in more detail. The current peaked a few milliseconds following the depolarizing voltage step, and inactivation appeared biphasic. A rapid initial phase lasted until 10 ms after the start of the voltage step, followed by a more slowly inactivating component (see inset in Figure 3A). In order to examine the kinetically different components of this outward current, we constructed IV-curves at three defined time points during the steps, i.e., for the peak of fast transient current (P), for current still active 10 ms after initiation of the depolarizing voltage step (P′), and for steady-state current at the end of the 1 s-long step (S; Figures 3B–D, respectively). Interestingly, only peak current amplitude (P) was different in AF compared to SR (significantly larger at +60 mV), whereas the IV-curves for P′ and S were not statistically different between AF and SR cells. As current declines over time during the clamp step, the apparent reversal potential shifted from about −60 mV (P), to near −20 mV at the end of the pulse (S), suggesting that—in addition to the rapidly activating and inactivating outward current component—there is a background (“leak”) current.

### Single BK_*Ca*_ Channel Currents, Cell-Attached and Inside-Out Modes

Since conflicting reports exist about the presence of BK_*Ca*_ currents in human atrial fibroblasts [compare Sheng et al. (2013) with Poulet et al. (2016)], we investigated whether currents with properties of BK_*Ca*_ were present in our cells. When magnifying the most depolarized portion of current traces activated by ramp pulses in whole-cell mode, one can notice single channel events (see Figure 4A).

To explore this further, we conducted recordings in inside-out and cell-attached configurations in SR cells. Current amplitudes in inside-out patches were voltage-dependent and enhanced upon increasing Ca^2+^ concentrations in the superfusate (facing the cytosolic side of the plasma membrane) from 0 to 0.1 mM, while the reversal potential of IV-curves was shifted to more negative potentials. This effect was reversible upon returning to nominally Ca^2+^-free solution (Figure 4B).

Also in inside-out patches (Figure 4C), channel activity in SR cells was completely blocked within 1 min of superfusion of the patch with the BK_*Ca*_ channel blocker paxilline (3 μmol L^–1^), and it was partially reversed after washout (representative recording shown, same effect observed on three cells isolated from two donors).

Ion channel activity (assessed in SR cells) was also abolished in cell-attached mode when the pipette solution contained paxilline (3 μmol L^–1^; (Zhou and Lingle, 2014) as shown in the traces for +40 and +60 mV in Figure 4D. In 21 fibroblasts from two donors, mean open probability was 0.0003 ± 0.0003 with paxilline in the pipette solution, compared to 0.026 ± 0.014 in 26 control cells from the same two donors.

Analysis of the data obtained in cell-attached voltage clamp mode (Figure 4E) revealed single channel activity whose open probability increased with increasingly positive clamp potentials. Open probability was smaller in AF than in SR fibroblasts [0.009 ± 0.003 (56 cells/4 AF patients) *vs.* 0.042 ± 0.012 (97 cells/7 SR patients) at +60 mV]. No statistically significant difference was seen between cells from SR and AF patients either in single channel conductance or in the percentage of cells that displayed such currents (Figure 4F). The presence of BK_*Ca*_ in atrial fibroblasts was confirmed by qPCR (Supplementary Figure 2A). In fibroblasts derived from a previous cohort of patients with AF and SR (Poulet et al., 2016), expression of *KNMA1* (BK_*Ca*_ subunit alpha1) was not different in cells from AF compared to SR patients (Supplementary Figure 2B).

Taken together these findings confirm the presence of BK_*Ca*_ channels in human right atrial fibroblasts, and their lower open probability in cells from AF, compared to SR tissue.

### Functional Role of BK_*Ca*_ Channels – Proliferation and Cell Shape

Some reports have related BK_*Ca*_ channel function in cardiac fibroblasts to proliferation and changes in cell shape (He K. et al., 2011; Sheng et al., 2013), while other groups did not find an effect of BK_*Ca*_ channel blockers on proliferation (Choi et al., 2008). In our hands, the absorbance (reporting the number of viable atrial fibroblasts: cells from five SR patients, analyses conducted in triplicate) increased by a factor of 1.8 ± 0.077 during 2 days of cell culture in control conditions (culture medium with 0.1% of DMSO as vehicle). This increase was significantly reduced, to 1.6 ± 0.077, when paxilline (10 μmol L^–1^) was added to the culture medium (Figures 5A,B).

Microscopic inspection of fibroblast cultures in control conditions or with paxilline (10 μmol L^–1^) suggested a paxilline-induced change in cell shape, from spindle-like to polygonal forms (Figures 5C,D). In order to quantify this change, we analyzed individual cell images for cell area and cell perimeter with ImageJ software and assessed circularity (see section “Materials and Methods”). For a perfect circular shape, the value of √A/P (circularity) will be at its maximum (=0.2821), while cells deviating from circular have smaller values. Circularity is larger in the presence of paxilline, confirming quantitatively the initial observation on cell shape differences (Figure 5E).

## Discussion

### Electrophysiology

Cultured human atrial fibroblasts, obtained by the outgrowth technique and used at passage 0 or 1, exhibit diverse patterns of voltage- and time-dependent currents, independent of whether they are derived from patients with SR or AF. In accordance with previous work (Poulet et al., 2016), we distinguished three types of rectification in steady-state currents: inward and/or outward, and no rectification. Neither the frequency of these three current patterns, nor their average IV-curves were different in cells derived from tissue donors with SR or AF. An inward rectifier current has been described in cardiac fibroblasts of both ventricular and atrial origin in several species (Chilton et al., 2005; Sheng et al., 2013; Aguilar et al., 2014; Qi et al., 2015; Poulet et al., 2016). Poulet et al. (2016) compared human atrial fibroblasts according to the patient’s pre-operative rhythm status and found that the inward rectifier current was significantly larger in AF than SR fibroblasts, which we did not observe in the present work.

We observed two voltage- and time-dependent transient outward currents with rapid and slower kinetics, as well as an inward current. The peak of the fast transient outward current had a linear IV-curve, and inactivation was almost complete after 10 ms. At +60 mV, peak current was significantly larger in AF than SR-derived fibroblasts. Fewer cells (20–60% less) showed slow outward currents, and no difference between fibroblasts derived from AF or SR patients was detected in this current.

Several groups have characterized inward currents in atrial and ventricular cardiac fibroblasts from rat and human as tetrodotoxin-sensitive Na^+^ currents (Walsh and Zhang, 2008; Li et al., 2009; Chatelier et al., 2012; Koivumaki et al., 2014; Poulet et al., 2016). In one study, I_*Na*_ was found in more cells from patients with AF (57% of 49) than from patients with SR (32% of 41) and peak I_*Na*_ was significantly larger in fibroblasts derived from AF patients (Poulet et al., 2016). Here, we observed inward currents in only 12% of AF and 24% of SR fibroblasts, without a statistically significant difference. Moreover, the voltage dependence of the observed (original) inward current (threshold, peak, and reversal at ≈−40, 0, and +60 mV, respectively) is not consistent with a fast Na^+^ current (Chatelier et al., 2012; Koivumaki et al., 2014; Poulet et al., 2016), but rather points toward L-type Ca^2+^ channels [see for instance Christ et al. (2004)]; this is an aspect that deserves further investigation.

BK_*Ca*_ channels have been reported previously in ventricular fibroblasts from rat (Choi et al., 2008) and human (Wang et al., 2006; Li et al., 2009; He M.L. et al., 2011). Interestingly, BK_*Ca*_ channel inhibition with paxilline markedly suppressed proliferation of human (He M.L. et al., 2011) but not of rat cardiac fibroblasts (Choi et al., 2008). In human atrial fibroblasts, BK_*Ca*_ channels were detected previously, but no information about heart rhythm was given (Sheng et al., 2013). Thus, the present study is the first report of lower BK_*Ca*_ channel activity in right atrial fibroblasts derived from AF compared to cells from SR donors. As *KCNMA1* mRNA expression is not altered in the context of AF, further experiments are needed to explore BK_*Ca*_ protein expression and localization, to determine whether the lower channel activity observed in AF compared to SR could be due to changes in protein trafficking and/or targeting to the plasma membrane for example. Also it will be important to include higher patient numbers to support robust statistical analyses.

Block of BK_*Ca*_ channel activity with paxilline alters cell shape of atrial fibroblasts and inhibits proliferation, as previously observed in ventricular fibroblasts (He M.L. et al., 2011). Since development and progression of AF is associated with structural remodeling and fibrosis (Burstein and Nattel, 2008), the involvement of BK_*Ca*_ channels in atrial fibroblast proliferation highlights these channels as target for further investigation into therapeutic interventions.

### Heterogeneity of Ion Currents in Human Atrial Fibroblasts

The recordings of ion currents in fibroblasts were surprisingly variable, which may in part be due to genuine diversity of ion channel expression and activity in cultured fibroblasts. Part of the variability in our results may be due to the presence of a mixed population of fibroblasts and myofibroblasts. In future work, it would be interesting to investigate the effect of fibroblast/myofibroblast phenoconversion on ion channel diversity and activity. This could be achieved by culture conditions enriching either phenotype.

Cells used here were cultured for an average duration of 25 days. Significant culture-related remodeling can occur in such a time-span, so that differences between fibroblasts from patients with SR or AF that were present originally *in vivo* may have leveled out. In this study, the fibroblasts analyzed are possibly pre-activated, as suggested by cell capacitance data which was in the range of 100 pF after an average time of 25 days in culture, both for SR and AF-derived fibroblasts, whereas (Poulet et al., 2016) reported such high capacitance values only in the AF group which have been more myofibroblastic [for comparison, (Sheng et al., 2013) found ≈40 pF for SR-derived fibroblasts]. The number of αSMA-positive cells has been assessed to be 17.6 ± 10.2% (*N* = 6) in cultures from SR patients (not shown); this percentage of αSMA-positive cells would be too small to explain the extent of variability in ion channel activities, suggesting a genuine intrinsic diversity of ion channel expression and/or function.

In summary, we confirm the presence of steady-state currents with different rectifying properties as well as voltage- and time-dependent outward currents in fibroblasts derived from patients with SR and in AF. We detect BK_*Ca*_ channels and find that they have a lower open probability in cells from AF, compared to SR patients. The different types of currents were observed in cells from the same donor tissue, showing that human cultured atrial fibroblasts are highly heterogeneous with respect to the pattern of ion channels they express.

## Data Availability Statement

The raw data supporting the conclusions of this article will be made available by the authors, without undue reservation.

## Ethics Statement

The studies involving human participants were reviewed and approved by Ethics Committee of Freiburg University, Nos 393/16 and 214/18. The patients/participants provided their written informed consent to participate in this study.

## Author Contributions

DJ, UR, PK, and RP contributed to conception, design, and interpretation of the study. DJ, AK, and ED performed and analyzed the experiments. FB and FAK provided access to surgical tissue samples. DJ, UR, and RP drafted the manuscript. All authors contributed to manuscript revision, read and approved the submitted version.

## Conflict of Interest

The authors declare that the research was conducted in the absence of any commercial or financial relationships that could be construed as a potential conflict of interest.

## Supplementary Material

Supplementary Figure 1 Capacitance of human atrial fibroblasts in primary culture. Capacitance of fibroblasts isolated from patients with SR and AF at passage 0 (average culture time 25 days, measured with the patch-clamp technique.

Supplementary Figure 2 *KCNMA1* mRNA Expression in human atrial fibroblasts from patients with SR and AF. **(A)** *KCNMA1* expression obtained by quantitative PCR in an immortalized cell line of human atrial fibroblasts (Künzel et al., 2020). *GAPDH* = glyceraldehyde-3-phosphate dehydrogenase, *n* = number of dishes analyzed corresponding to three independent experiments. **(B)** Mean *KCNMA1* expression from the Affymetrix GeneChip array, performed in the study by Poulet et al. (2016) on atrial fibroblasts in primary culture isolated from patients with SR and AF, *de novo* analysis of original data from Poulet et al. (2016). Analysis performed after one passage (5 weeks of culture). N: number of patients; A.U.: arbitrary units.

Supplementary Table 1 Ion Currents/Channels in Cardiac Fibroblasts. CM, cardiomyocytes; FB, fibroblasts; LA, left atrium, LV, left ventricle; CHF, congestive heart failure; MI, myocardial infarction; TEA, tetraethylammonium chloride; 4-AP, 4-Aminopyridine; →: leaded to; ↑: increase; ↓: decrease.
