# Supplementary figures and images for: Heterogeneity and Remodeling of Ion Currents in Cultured Right Atrial Fibroblasts From Patients With Sinus Rhythm or Atrial Fibrillation
Source: Front Physiol. 2021 Jun 3;12:673891. doi: 10.3389/fphys.2021.673891 (PMC8209389; doi:10.3389/fphys.2021.673891)

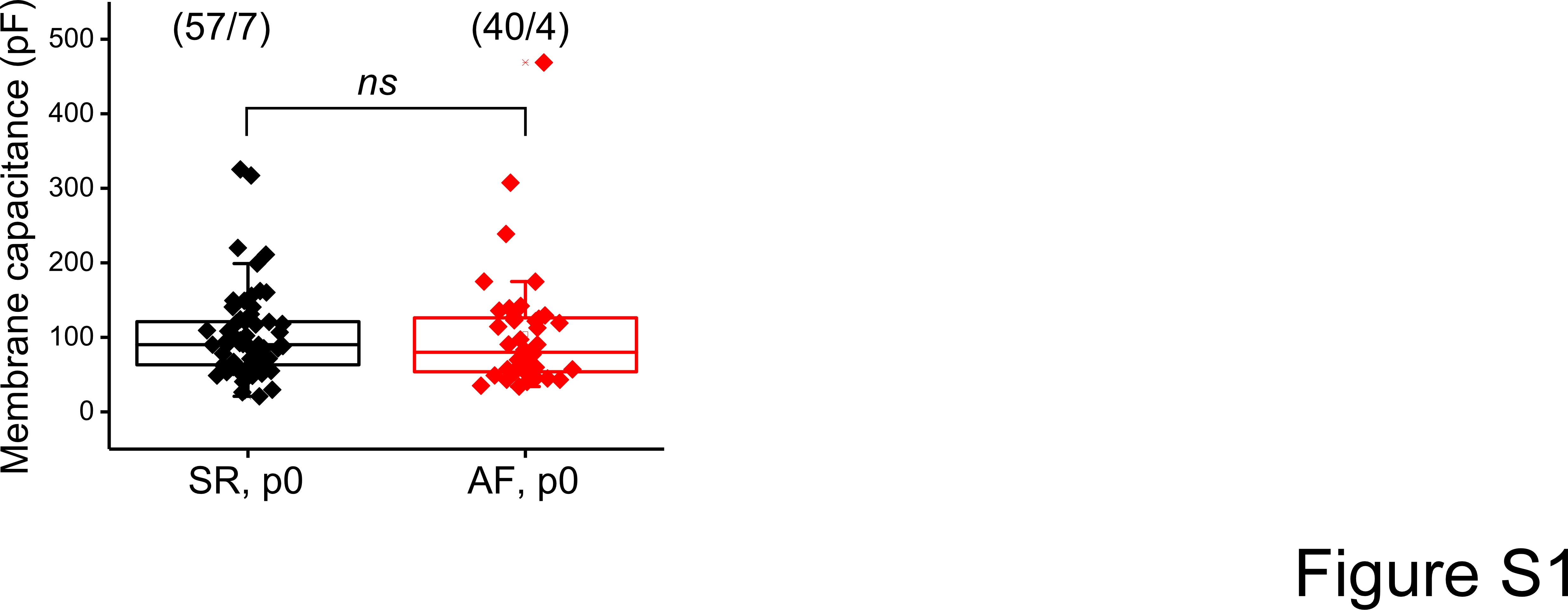

Supplement: Supplementary Figure 1 — Capacitance of human atrial fibroblasts in primary culture. Capacitance of fibroblasts isolated from patients with SR and AF at passage 0 (average culture time 25 days, measured with the patch-clamp technique. [file Image_1.jpg]

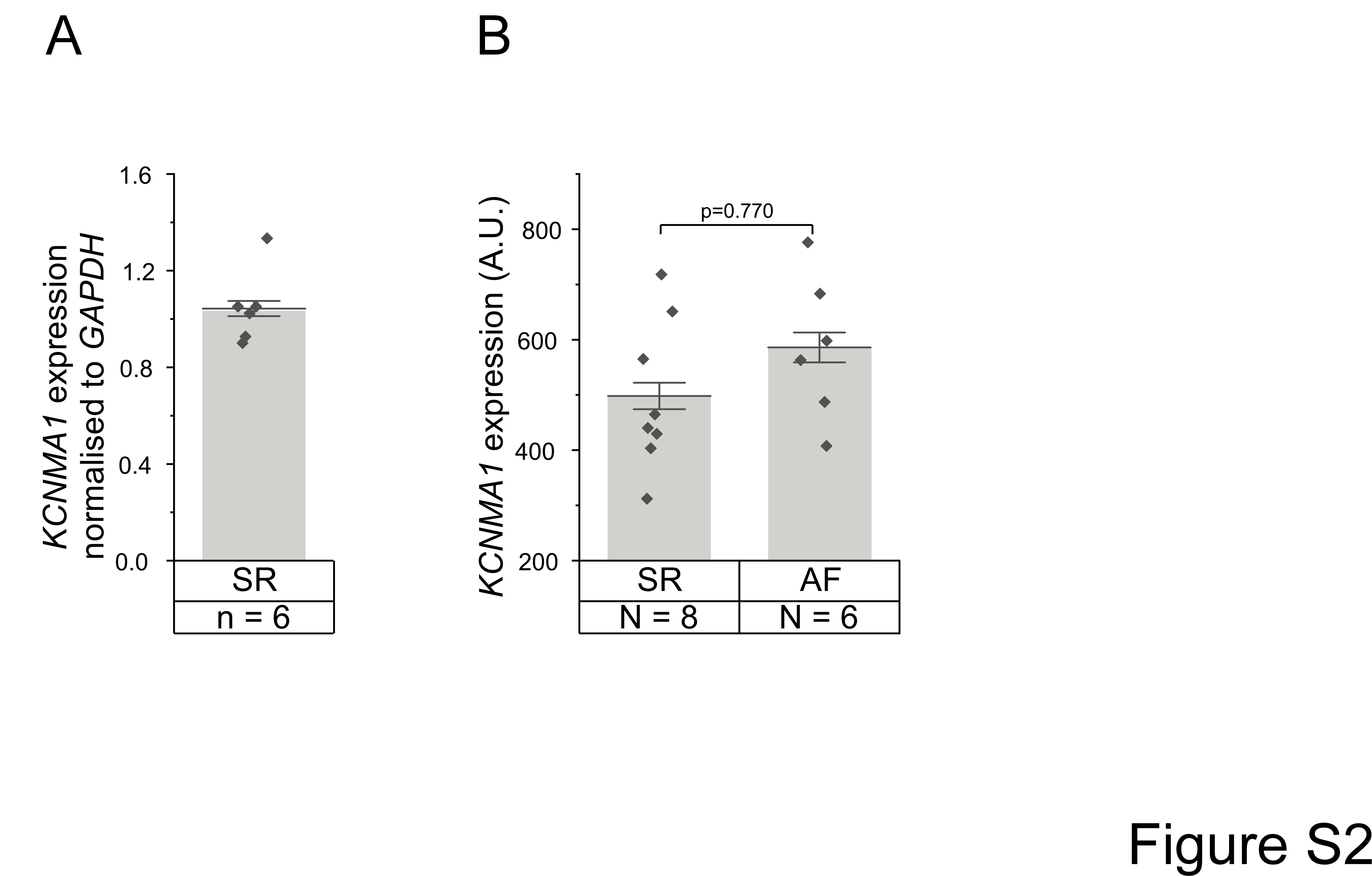

Supplement: Supplementary Figure 2 — KCNMA1 mRNA Expression in human atrial fibroblasts from patients with SR and AF. (A) KCNMA1 expression obtained by quantitative PCR in an immortalized cell line of human atrial fibroblasts (Künzel et al., 2020). GAPDH = glyceraldehyde-3-phosphate dehydrogenase, n = number of dishes analyzed corresponding to three independent experiments. (B) Mean KCNMA1 expression from the Affymetrix GeneChip array, performed in the study by Poulet et al. (2016) on atrial fibroblasts in primary culture isolated from patients with SR and AF, de novo analysis of original data from Poulet et al. (2016). Analysis performed after one passage (5 weeks of culture). N: number of patients; A.U.: arbitrary units. [file Image_2.JPEG]
