# Supplementary material for: Heterogeneity and Remodeling of Ion Currents in Cultured Right Atrial Fibroblasts From Patients With Sinus Rhythm or Atrial Fibrillation
Source: Front Physiol. 2021 Jun 3;12:673891. doi: 10.3389/fphys.2021.673891 (PMC8209389; doi:10.3389/fphys.2021.673891)
Supplement: Supplementary Table 1 — Ion Currents/Channels in Cardiac Fibroblasts. CM, cardiomyocytes; FB, fibroblasts; LA, left atrium, LV, left ventricle; CHF, congestive heart failure; MI, myocardial infarction; TEA, tetraethylammonium chloride; 4-AP, 4-Aminopyridine; →: leaded to; ↑: increase; ↓: decrease. [file Table_1.docx]

**Supplementary Table 1: Ion Currents/Channels in Cardiac Fibroblasts**

CM: cardiomyocytes; FB: fibroblasts; LA: left atrium, LV: left ventricle; CHF: congestive heart failure; MI: myocardial infarction; TEA: Tetraethylammonium chloride; 4-AP: 4-Aminopyridine; 🡒: leaded to; ↑: increase; 🡓: decrease.

| **Species** | **Fibroblasts** | | **Current** | **Channel Protein** | **Function / Alteration in AF / models of AF** | **Reference** |
| --- | --- | --- | --- | --- | --- | --- |
|  | **Origin** | **Isolation** |  |  |  |  |
| **I_to_** | | | | | | |
| Rat (neonatal) | Whole heart | Enzymatic isolation plus 24-48 h culture | I_to_ | - | (Emphasis of paper on CM-FB and FB-FB coupling) | Rook et al. 1992 |
| Rat (neonatal) | Ventricle | Primary culture: passage 2 (days 3-4) | I_to_  I_Kf_  I_Ks_ | Kv1.4  Kv1.5, Kv1.2  Kv2.1 | In 58% of cells (of a total of 67 cells)  In 22%    In 19%  (no Ba^2+^-sensitive inward rectifier) | Walsh & Zhang 2008 |
| Man | Ventricular | Cultured, commercially available | I_to_ | Kv4.x | Detected in 15% of cells | Li G-R et al. 2009 |
| Man | Atria | Cultured (outgrowth) passage 2-3 | I_to_ | Kv4.3 | 4-AP-sensitive current in 34% of cells (n=309); [current density 🡓with H_2_S, IC_50_ 55.1 µM] | Sheng J et al. 2013 |
| Man | Right atria | Freshly isolated, cultured (outgrowth) | I_to_ | Kv4.1 | Reduced expression of Kv4.1 in AF FB | Poulet et al. 2016 |
| Dog, tachypacing-induced CHF | LV, LA | Freshly isolated | I_to_ | Kv4.3 | Kv4.3: LA < LV, CHF 🡓;  Inhibition of Kv current with TEA 🡒 proliferation 🡑 | Wu CT et al., 2014 |
| **I_Kv_** | | | | | | |
| Rat | Ventricle | Freshly isolated (used within 6 h) | I_Kv,TEA_ | - | Time and voltage-dependent K current; Sensitive to dendrotoxin-I (100 nM) and rTityustoxin Kα (50 nM) | Shibukawa et al. 2005 |
| Dog, tachypacing-induced CHF | Left atria | Freshly isolated, cultured up to 15 days | I_Kv,TEA_ | - | Down-regulated within 48 h of culture (activtion of FB into MFB) | Dawson et al. 2012 |
| Dog, tachypacing-induced CHF | Left atria | Freshly isolated | I_Kv,TEA_  (I_to_/I_Kur_) | Kv4.3, Kv1.5 | Down-regulated in AF | Aguilar et al. 2014 |
| Man | Ventricle | Cultured, commercially available | I_Kur_ | Kv1.5 | Detected in 14% of cells | Li G-R et al. 2009 |
| Dog, tachypacing-induced CHF | LV, LA | Freshly isolated | I_Kur_ | Kv1.5 | Kv1.5: LA < LV, CHF 🡓;  Inhibition of Kv current with TEA 🡒 proliferation 🡑 | Wu CT et al. 2014 |
| Man | Right atria | Cultured (outgrowth) | I_Ks_ | Kv7.1 | Very low expression | Poulet et al., 2016 |
|  |  |  | I_Kr_ | Kv11.1 | Unknown |  |
| **Proton current** | | | | | | |
| Man | Right atria | Cultured up to 6 days only | I_H_ | Proton channel | Delayed outward current, H^+^ selective; Voltage-gated proton channel, dependent on internal pH, Zn^2+^ sensitive | El Chemaly et al. 2006 |
| **I_Kir_** | | | | | | |
| Rat | Ventricle | Freshly isolated / cultured | I_Kir_  I_to_ | Kir2.1  Kv1,6 | Hyperpolarisation 🡒 proliferation 🡑  Depolarisation 🡒 proliferation 🡓 | Chilton et al. 2005 |
| Man | Atria | Cultured (outgrowth) passage2-3 | I_Kir_ | - | Ba^2+^-sensitive current in 28% of cells (n=309); [current density 🡓 with 100 µM H_2_S] | Sheng J et al. 2013 |
| Dog, tachypacing-induced CHF | Left atria | Freshly isolated | I_K1_ | Kir2.1, Kir2.3 | Upregulated in AF | Aguilar et al. 2014 |
| Dog, tachypacing-induced CHF | Left atria | Freshly isolated | I_K1_ | Kir2.1, Kir2.3 | Upregulation in CHF 🡒 store-operated Ca^2+^ entry 🡑, proliferation 🡑 | Qi XY et al. 2015 |
| Man | Right atria | Freshly isolated, cultured (outgrowth) | I_K1_ | Kir2.1, Kir2.3 | Detected; larger currents in cultured FB | Poulet et al., 2016 |
| **I_K,ACh_** | | | | | | |
| ? |  |  | I_K,ACh_ | Kir3.1/3.4 | Unknown |  |
| **I_K,ATP_** | | | | | | |
| Mouse | Ventricle | Culture (passage?) |  | SUR2/ Kir6.1 | Channels stimulated by sphingosine-1-phosphate; increase in S1P-induced current with culture time (day 5-9); proliferation 🡑; collagen production 🡓 | Benamer et al. 2011 |
| Rat | Ventricle | Freshly isolated (4 d) | I_K,ATP_ | SUR2/ Kir6.1 | No pinacidil and glibeclamine-sensitive I_KATP_ absent in normal FB (normal hearts, remote MI zone), but I_K,ATP_ in scar and boarder zone MI (myofibroblasts); modulate proliferation | Benamer et al. 2013 |
| ? | Atria |  | I_KATP_ | Kir6.1 | Unknown |  |
| **BK_Ca_ channels** | | | | | | |
| Rat | Ventricle | Culture (enzym-atic dissociation) passage 3 | I_K,Ca_ | BK  IK  SK1-4 | Iberiotoxin (0.1 µM) – proliferation no effect  Clotrimoxazol (2 µM) – proliferation no effect  Apamin (0.5 µM) proliferation 🡑 (to 116.6%) | Choi S et al. 2008 |
| Man | Ventricle | Cultured, commercially available | I_K,Ca_ | BK, K_Ca_1.1 | Detected | Wang Y-J et al. 2006 |
| Man | Ventricle | Cultured, commercially available | I_K,Ca_ | BK, K_Ca_1.1 | Detected in 88% of FB | Li G-R et al. 2009 |
| Man | Ventricle | Culture (enzym-atic dissociation) passage 3-6 | I_K,Ca_ | BK, K_Ca_1.1 | Paxilline-sensitive current  Proliferation 🡓 by paxillin (0.1-3 µM) | He et al. 2011 |
| Man | Atria | Cultured (outgrowth) passage 2-3 | I_K,BK_ | BK, K_Ca_1.1 | Paxilline-sensitive current in 52% of cells (n=309); [current density 🡓 with H_2_S, IC_50_ 69.4 µM] | Sheng J et al. 2013 |
| Dog, tachypacing-induced CHF | Left ventricle, left atria | Freshly isolated | I_K,Ca_ | BK, K_Ca_1.1 | BK_Ca_: LA > LV, CHF no effect  Inhibition of KCa1.1 with paxilline 🡒  proliferation 🡑 | Wu CT et al. 2014 |
| Man | Right atrium | Cultured (outgrowth) passage 0-1 | I_K,Ca_ | BK | (preliminary results) | Klesen et al. 2018 |
| **I_Na_** | | | | | | |
| Rat (neonatal) | Ventricle | Primary culture: passage 2 (days 3-4) | I_Na,TTX_ | - | In 36% of cells (of a total of 67 cells) | Walsh & Zhang 2008 |
| Man | Ventricle | Cultured, commercially available | I_Na,TTX_ | Nav1.7 Nav1.5 Nav1.2 Nav1.3 Nav1.6 | I_Na_ in 63% of cells | Li G-R et al. 2009 |
| Man | Atria | Cultured (12 d) | I_Na_ | Nav1.5 | Appear upon differentiation into MyoFB | Chatelier et al. 2012 |
| Man | Atria | Cultured 6 d (passage 2-4) | I_Na_ | Nav1.5 Nav1.9 Nav1.2 | 40-50% of ‘MyoFB’ express I_Na_ | Koivumaki et al. 2014 |
| Man | Right atria | Cultured | I_Na_ | Nav1.5 | TTX sensitive I_Na_ in 57% of AF (n=49) and 32% of SR cells (n=41); peak I_Na_: AF > SR | Poulet et al., 2016 |
| **I_CRAC_** | | | | | | |
| Man | Left ventri-cle (LVAD) | Cultered (out-growth), used at passage 2-3 | I_CRAC_ | SOCE, Orai/STIM | Thapsigargin-depleted Ca^2+^ stores 🡒 increase in [Ca^2+^]_e_ for SOCE (maximum influx tested with ionomycin) | Ross et al. 2017 |
| **I_Cl_** | | | | | | |
| Man | Ventricle | Cultured, commercially available | I_Cl_ | Clnc3 |  | Li G-R et al. 2009 |
| Man | Ventricle | Culture (enzym-atic diss.) passage 3-6 | I_Cl_ | Clnc3 | DIDS-sensitive current  Proliferation 🡓 by DIDS (100-200 µM) | He ML et al. 2011 |
| Rat, neonatal | Left ventricle | Cultured (passage 3) | I_Cl,Ca_ |  | Cl^-^ influx into nucleus: inhibition affects proliferation | Tian X et al. 2018 |
| **TRP channels** | | | | | | |
| Man | Right atria | Freshly isolated |  | TRPM7 | Upregulated in RAApp-FB from AF patients; major Ca^2+^ entry pathway | Du J et al. 2010 |
| Rat, Dog, Goat, Man | Left atrium; RAApp | Freshly isolated | I_NS,cat_ | TRPC3 | Pyrazol-3- and Gd^3+^-sensitive, non-voltage gated, non-selective cation current; up-regulation in AF | Harada et al. 2012 |
| **Mechano-sensitive channels** | | | | | | |
| Rat | atria | Freshly isolated | Cation nonselect. | ? | Blocked by Gd^3+^, cytochalasin D and colchicine | Kamkin et al. 2010 |
| Mouse, human | atria | cutlured | Cation nonselect. | Piezo1 | Regulation of interleukin-6 secretion | Blythe et al. 2019 |
